# Identification of Sestrin3 Involved in the *In vitro* Resistance of Colorectal Cancer Cells to Irinotecan

**DOI:** 10.1371/journal.pone.0126830

**Published:** 2015-05-14

**Authors:** Seung Ho Choi, Hye Kyung Hong, Yong Beom Cho, Woo Yong Lee, Hae Yong Yoo

**Affiliations:** 1 Department of Health Sciences and Technology, Samsung Advanced Institute for Health Sciences and Technology, Sungkyunkwan University, Seoul, Korea; 2 Samsung Biomedical Research Institute, Research Institute for Future Medicine, Samsung Medical Center, Seoul, Korea; 3 Department of Surgery, Samsung Medical Center, Sungkyunkwan University School of Medicine, Seoul, Korea; University of Navarra, SPAIN

## Abstract

Irinotecan, an analogue of camptothecin, is frequently used as a single agent or in combination with other anticancer drugs for the treatment of colorectal cancer. However, the drug resistance of tumors is a major obstacle to successful cancer treatment. In this study, we established that cells acquire chronic resistance to irinotecan. We profiled their differential gene expression using microarray. After gene ontology (GO) and KEGG pathway analysis of the microarray data, we specifically investigated whether Sestrin3 could decrease irinotecan resistance. Our results revealed that Sestrin3 enhanced the anticancer effect of irinotecan *in vitro* in LoVo cells that had acquired resistance to irinotecan. Irinotecan-resistant LoVo cells showed lower reactive oxygen species (ROS) production than their irinotecan-sensitive parental cells. ROS production was increased by Sestrin3 knockdown in irinotecan-resistant LoVo cells. Our results indicate that Sestrin3 might be a good target to develop therapeutics that can help to overcome resistance to irinotecan.

## Introduction

The incidence of colorectal cancer is over one million per year worldwide, with a mortality rate of up to 33% in developed nations [1]. Colorectal cancer is the fifth most common cancer. Chemotherapy has been recognized as being effective in treating metastasized colorectal cancer. Typically, fluorouracil (5-FU) has been used as a single therapy. However, in the last two decades, clinical practice has been using cytotoxic drugs such as fluoropyrimidine, irinotecan, and oxaliplatin. Standard combination chemotherapy regimens are FOLFIRI (folinic acid, fluorouracil, and irinotecan), CapIri (capecitabine and irinotecan), FOLFOX (folinic acid, fluorouracil, and Oxaliplatin), or CapOx (capecitabine and oxaliplatin). Substituting irinotecan with oxaliplatin has contributed to improved survival rate [2, 3]. Irinotecan (CPT-11), a derivative of natural camptothecin, is a major therapeutic drug for metastatic colorectal cancer (CRC) patients [4]. Irinotecan is chemically converted to its active form, 7-ethyl-10-hydroxycamptothecin (SN-38), which inhibits DNA topoisomerase. The stalling of topoisomerase at the replication fork by SN-38 induces a permanent DNA double-stranded break, which produces a DNA damage response (DDR). DNA damage is primarily sensed by the kinases ATR and ATM, the increased activity of which leads to the activation of the checkpoint kinases chk1/chk2 and the subsequent phosphorylation of p53. Phosphorylated p53 is more stable, which can activate apoptosis or regulate cell cycle arrest. p53 also plays a role in antioxidant response, which was discovered through the identification of a novel Sestrin (*SESN*) gene family, which is involved in reactive oxygen species (ROS) regulation [5]. Based on primary and secondary structure analysis using the PSI-BLAST and 3DPSSM programs, the *SESN* family is reported to encode antioxidant proteins [6, 7]. Sestrin proteins have a high degree of homology with the *Mycobacterium tuberculosis* protein AhpD, sharing similarities in their N-terminal domains [5]. They are responsible for catalyzing the reduction of peroxiredoxins (Prdx) that metabolize peroxides [8]. The AhpD protein, a component of alkyl-hydroperoxide reductase participating in the defense against ROS, is responsible for the regeneration of AhpC, a member of a conserved family of thiol-specific peroxidases (Prxs) [9]. The *Sestrin1* and *Sestrin2* genes are transcriptionally regulated under the control of p53, whereas *Sestrin3* is regulated by the AKT/FOXO axis, through FOXO1/FOXO3a-mediated gene expression [5, 10]. *Sestrin3* is also involved in ROS detoxification as well as in delaying cellular senescence through FOXO [11]. Of the three members of the Sestrin family, the third member, *Sestrin3*, has been reported in the literature to a lesser extent than the two others.

Although irinotecan displays potent activity against a wide range of tumors, including colorectal tumors, drug resistance remains a major obstacle to effective chemotherapy. Therefore, novel targets to overcome drug resistance are needed for successful cancer treatment. Several hypotheses have been proposed regarding the mechanisms involved in the resistance to CPT, including reduced cellular accumulation of CPT due to active efflux by ATP binding cassette (ABC) transporters, enzymatic systems relevant to metabolic conversion, alteration in the structure or location of topoisomerase I, and alterations in the cellular response to CPT-TOPI-DNA ternary complex formation [7]. Although multiple experimental approaches have been attempted clinically to overcome individual mechanisms of resistance [7], remarkable success has yet to be achieved.

In this study, we established a cell line that acquired chronic resistance to irinotecan. Our objective was to compare the transcriptional profile in the irinotecan-resistant colorectal cancer cell line to that in the parental irinotecan-sensitive cells, to search for new markers to increase the sensitivity to irinotecan. We also investigated whether Sestrin3 could enhance the anticancer effect of irinotecan *in vitro*, in the irinotecan-resistant colorectal cancer cell line.

## Materials and Methods

### Cell lines and culture conditions

The human colorectal cancer cell lines HCT116, HCT15, HCT29, KM12SM, SW620, DLD1, CoLo205, and LoVo were obtained from the American Type Culture Collection (ATCC). Cell lines were cultured in RPMI 1640 supplemented with 10% fetal bovine serum (FBS), 100 U/ml penicillin, 100 μg/ml streptomycin, 1 mM sodium pyruvate, and 2.05 mM L-glutamine at 37°C. Cell lines were regularly tested for identity and mycoplasma infection, using the MycoAlert mycoplasma detection kit (Lonza).

### Western blot analysis

Cells were lysed in RIPA buffer (50 mM Tris, 150 mM NaCl, 1% NP-40, 0.5% sodium deoxycholate, and 0.1% sodium dodecyl sulfate) supplemented with protease inhibitors and phosphatase inhibitors. Antibodies used for immunoblotting were rabbit monoclonal anti-ATM (D2E2, Cell signaling), rabbit polyclonal anti-phospho-Chk1 (Ser317, Cell signaling), rabbit polyclonal anti-ATR, TopBP1, CtIP (Bethyl laboratories), and mouse monoclonal anti-NBS1 (1D7, Novus Biologicals). Polyclonal antibodies against Sestrin3 were purchased from Abcam. Mouse monoclonal anti-Chk1 (G-4) and anti α-tubulin were purchased from Santa Cruz biotechnology. Antibodies against AMPKα (23A3), phospho-AMPKα (Thr72, 40H9), p70S6K and phosphor-p70S6K (Thr389, 108D2), mTOR (7C10), and phospho-mTOR (Ser2448) were purchased from Cell Signaling.

### Development of the irinotecan-resistant cell line

To create a stable colorectal cancer cell line chronically resistant to irinotecan, LoVo cells were exposed to irinotecan at an initial concentration of 0.1 μmol/l in RPMI 1640 supplemented with 10% FBS. When cultured cells reached a confluency of 80%, 20% of the cells were replated for next passage. Cells were passaged 3 times at the same concentration of irinotecan to ensure their adaptation and then treated with 2 fold higher concentration of irinotecan. The concentration of irinotecan was sequentially increased until it reached a final concentration of 8 μmol/l. Irinotecan was purchased from Sigma-Aldrich.

### Cell Viability and cytotoxicity assays

Colorectal cancer cell lines were seeded into 96-well cell culture plates in RPMI 1640, supplemented with 10% FBS. After 24 h of incubation, the medium was replaced with fresh medium containing irinotecan. Various concentrations of irinotecan were used to treat cells for 72 h. The cell viability or cytotoxicity was evaluated using the Cell Counting Kit-8 (CCK-8) assay, according to the manufacturer’s instructions. Briefly, 10 μl of CCK-8 solution was added to each well. Plates were incubated for 2 h at 37°C. The absorbance values were measured at 450 nm.

### Clonogenic survival assay

Cells were cultured in a medium containing irinotecan at concentrations of 0.05, 0.1, 0.4, 0.8, 1.5, and 3 μM, respectively. After 24 h, the cells were detached and seeded onto 60-mm culture dishes. After 14 days, the remaining colonies were washed with PBS, stained with crystal violet, and then counted according to defined colony sizes. All experiments were independently repeatedly at least 3 times. The statistical significance of the difference in colony numbers was determined using a two-tailed Student’s t-test.

### Cell cycle analysis

For cell cycle analyses, flow cytometry was carried out using LoVo cells treated with or without irinotecan. Briefly, cells were detached with trypsin, washed with PBS, and resuspended in 0.5 ml of PBS. After fixation with 4.5 ml of 70% ethanol, cells were incubated on ice for 2 h. Fixed cells were washed, resuspended in 1 ml of a propidium iodide staining solution containing 0.1% (v/v) triton X-100, 0.2 mg/ml of RNase A, and 20 μg/ml of PI, incubated at room temperature for 30 min, and then kept on ice until the flow cytometry analysis was performed.

### RNA Interference (RNAi)

For transient RNA silencing, cells were transfected with siRNA-targeting Sestrin3 (siSESN3) or with non-targeting siRNA (siControl), using the lipofectamine RNAiMAX transfection reagent (Life Technologies).

### Measurement of cellular ROS

The generation of intracellular ROS was evaluated using the CellRox green oxidative stress reagent (Invitrogen). Briefly, cells were transfected with siRNAs for 48 h and subsequently incubated with 5 μM of the CellRox reagent at 37°C for 30 min. After washing twice with PBS, the fluorescence intensity was measured using fluorescent microscopy.

### RNA Isolation and Gene Expression Profiling

In the present study, we performed global gene expression analyses using Affymetrix GeneChip Human Gene 1.0 ST oligonucleotide arrays using LoVo cells and irinotecan resistant LoVo-R8 cells cultured with or without irinotecan (8 μM). Samples were prepared according to the instructions and recommendations provided by the manufacturer. The total RNA was isolated using RNeasy Mini Kit columns (Qiagen). The RNA quality was assessed using the Agilent 2100 bioanalyzer with an RNA 6000 Nano Chip (Agilent Technologies). The RNA quantity was determined using the ND-1000 Spectrophotometer (NanoDrop Technologies, Inc.). RNA samples (300 ng each) were used as input for the Affymetrix procedure as described in the protocol. Briefly, 300 ng of total RNA from each sample was converted to double-stranded cDNA, using a random hexamer incorporating a T7 promoter. Amplified RNA was generated from the double-stranded cDNA template though *in vitro* transcription and purified with the Affymetrix sample cleanup module. cDNAs were regenerated by reverse transcription using random primers and a dNTP mix containing dUTP. cDNAs were then fragmented by UDG and APE 1 restriction endonucleases and end-labeled by a terminal transferase reaction that incorporated a biotinylated dideoxynucleotide. Fragmented and end-labeled cDNAs were hybridized using the GeneChip Human Gene 1.0 ST arrays for 16 hours at 45°C and 60 rpm, as described in the Gene Chip Whole Transcript (WT) Sense Target Labeling Assay Manual (Affymetrix). After hybridization, chips were stained and washed in the Genechip Fluidics Station 450 (Affymetrix), and scanned using a Genechip Array scanner 3000 7G (Affymetrix). For statistical analysis, a detection call (Present/Absent) was generated by the Affymetrix microarray suite 5(MAS5) algorithm. The scanned raw files were imported into the statistical programming environment R (Version2.3), for further analysis with tools available from the Bioconductor Project. Expression data were normalized and log2-transformed, using the robust multichip average (RMA) method implemented in the Bioconductor package RMA (M2, M3). To reduce noise in the significance analysis, probe sets that did not show a detection call rate of at least 50% of the samples were filtered out. Highly expressed genes that showed a 2-fold change in expression were selected. Results were classified using hierarchical clustering algorithms implemented in the TMEV software 4.0. Array data were deposited at the Gene Expression Omnibus with the accession number GSE59501.

### Statistical analysis

*In vitro* experimental data were obtained from experiments repeated three times in triplicates. Mean values were calculated, and significance was determined, using the Student’s two-tailed test. *P* values < 0.05 were considered statistically significant.

## Results

### Establishment of irinotecan-resistant cell lines

**Fig 1:**
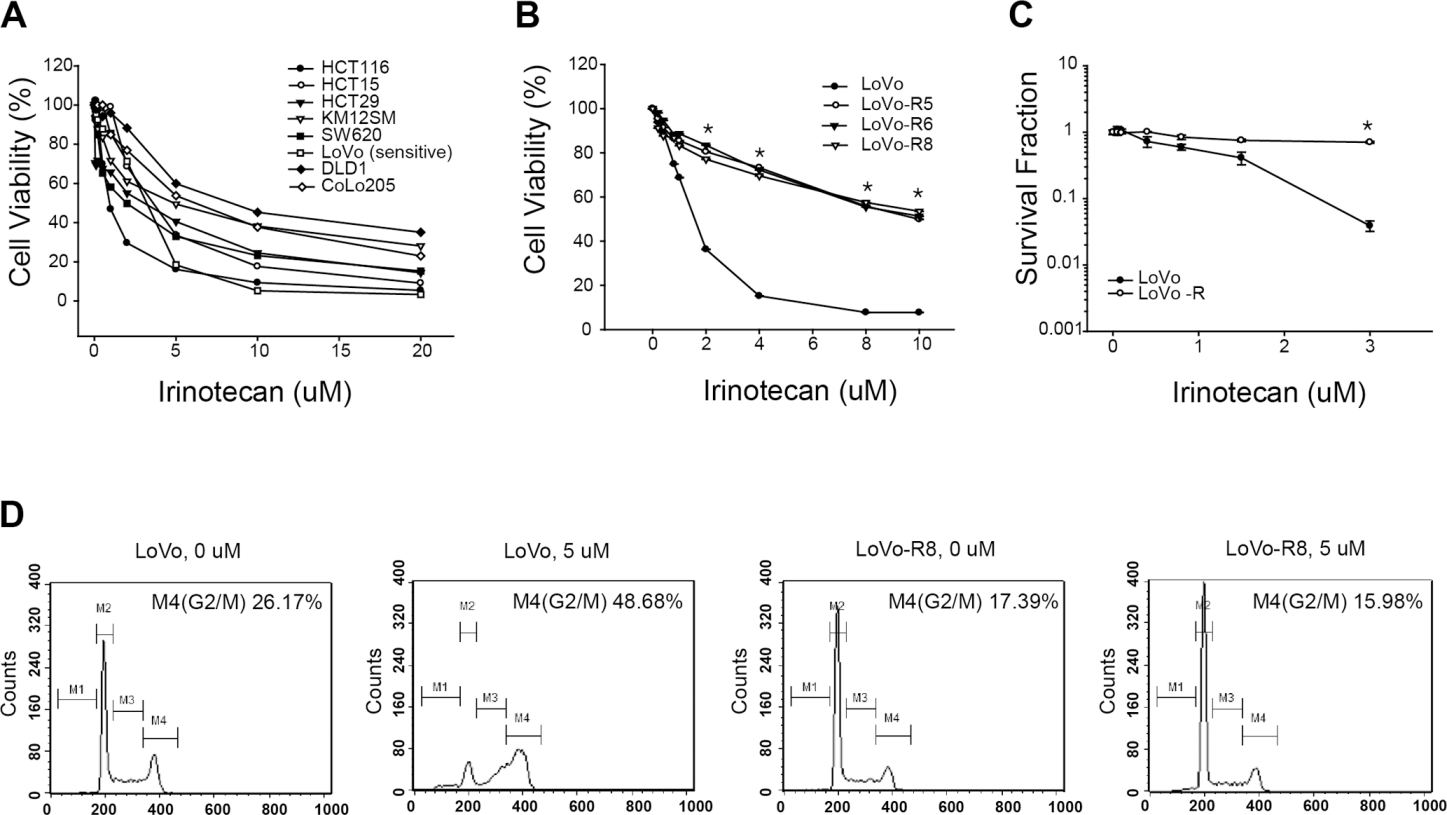
Effect of irinotecan on cell proliferation in colon cancer cells. (A) The sensitivity of eight colon cancer cell lines to irinotecan was measured using the CCK-8 assay. For the CCK-8 assay, cells were exposed to irinotecan at given concentrations for 72 h before measurement. The cell viability was presented as the percentage relative to untreated cells. (B) The resistance of established LoVo cells to irinotecan was measured using the CCK-8 assay and (C) the clonogenic survival assay. Colonies that survived the clonogenic survival assay were measured after further incubation for an additional 14 days. (D) Cell cycle progression of LoVo-R8. * *p* ≤ 0.05.

Before generating a colon cancer cell line with acquired resistance to irinotecan, we tested the cytotoxicity of irinotecan on several colorectal cancer cell lines to identify the most sensitive one. Of eight cell lines, the LoVo cell line was the most sensitive to irinotecan (Fig 1A). The cytotoxicity of irinotecan to parental and resistant LoVo (LoVo-R) cells was determined using the CCK-8 assay. The LoVo-R cells were more resistant to irinotecan than the parental LoVo cells. The IC_50_ values of LoVo-R and parental LoVo were 10 μM and 1.5 μM, respectively. We established several resistant cell lines using different final concentrations of irinotecan. However, similar levels of IC_50_ were found across the resistant cell lines with different final concentrations of irinotecan (Fig 1B). The resistance of the LoVo-R8 cell line was confirmed using a clonogenic assay (Fig 1C). The LoVo-R8 cell line adapted to irinotecan at a final concentration of 8 μM. It is well known that irinotecan specifically inhibits DNA replication by trapping topoisomerase I in DNA strands, inducing a cell cycle arrest in the G_2_ phase. To investigate the effect of irinotecan on the cell cycle progression of LoVo-R8 cells, we further analyzed the cell cycle of this cell line (Fig 1D). The LoVo parental cells that were treated with 5 μM of irinotecan showed severe G_2_/M arrest. After 24 h of irinotecan treatment (5 μM), the cell cycle arrest at the G_2_/M-phase was seen only in the parental LoVo cells, but the LoVo-8R cells treated with or without irinotecan showed no difference in their cell cycle progression.

### Gene expression array analysis of irinotecan-resistant LoVo-R8 cells

**Fig 2:**
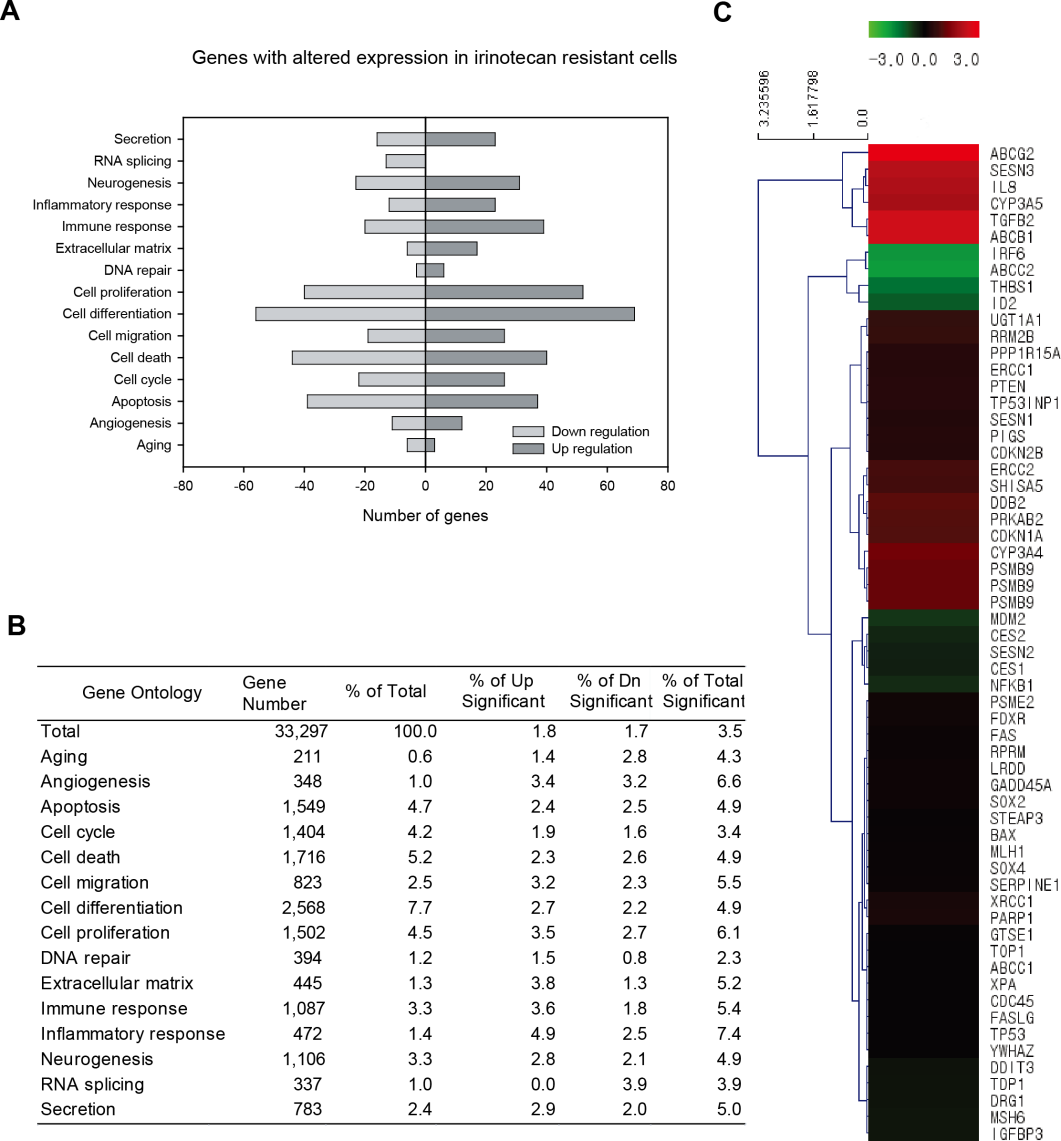
Gene ontology-based expression analysis in irinotecan-resistant LoVo cells, compared to their parental cells. Genes were selected using a filter criterion of at least a 2-fold change compared to controls with *p* < 0.05. Genes with altered expression in the irinotecan-resistant LoVo cell line, compared to the original LoVo cell line, are categorized into 15 functional groups based on gene ontology. The gene numbers are displayed in graphs (A) and probed gene numbers in this array analysis and percentage values of the significantly changed genes are presented in a table (B). (C) Hierarchical cluster analysis of the irinotecan-resistant LoVo cell expression microarrays. A cluster-based representation of altered genes in irinotecan-resistant cells with intensity, normalized to the parental LoVo cell line. Genes that were up-regulated relative to parental LoVo cells are shown in red, and those that were down-regulated are shown in green. The expression levels of these genes were altered ≥1.5-fold or ≤0.6-fold in irinotecan-resistant LoVo cell lines, compared with the original LoVo cell line. Gene symbols are shown in the right row.

After establishing the irinotecan-resistant LoVo-8R cell lines, we investigated their gene expression profiles using DNA microarray. Using a filter criterion of at least a 2-fold change with *p* < 0.05, the number of genes with changes in expression in LoVo-8R cells, compared to their parental LoVo cells, was determined. A total of 599 and 566 genes were up-regulated and down-regulated, respectively, accounting for 3.5% of the total genes probed (Fig 2A and 2B). A functional annotation of these genes was carried out, using a gene ontology-based analysis of biological properties. The genes were categorized into 15 functional groups (Fig 2A and 2B). Most of these genes were associated with the inflammatory response, angiogenesis, cell proliferation, or cell migration.

A hierarchical clustering heat map (Fig 2C) depicts the patterns of change in gene expression levels observed in the irinotecan-resistant LoVo cells. A total of 24 genes were shown to be involved in the irinotecan pathway or to be cell cycle arrest-related genes, based on the analysis of biological properties conducted using gene ontology. A total of 53 pathways were found, based on the KEGG pathway analysis. The entire clustering heat map is presented as supplemental data in S1 Fig. We also analyzed the functions of known genes involved in the irinotecan pathway of colorectal cancer cells. CYP3A4 and CYP3A5 are involved in the formation of carbonyloxycamptothecin, an inactive metabolite of irinotecan. The RNA levels of two ATP-binding cassette transmembrane proteins (ABC), ABCG2 (known as breast cancer resistance protein) and ABCB1, were dramatically up-regulated in the irinotecan-resistant cells (Fig 2C and S1 Table). This is not surprising, considering the fact that efflux of irinotecan in tumor cells is revealed as a potential strategy to overcome drug resistance.

### Expression of genes involved in the pathway of DNA damage checkpoint

**Fig 3:**
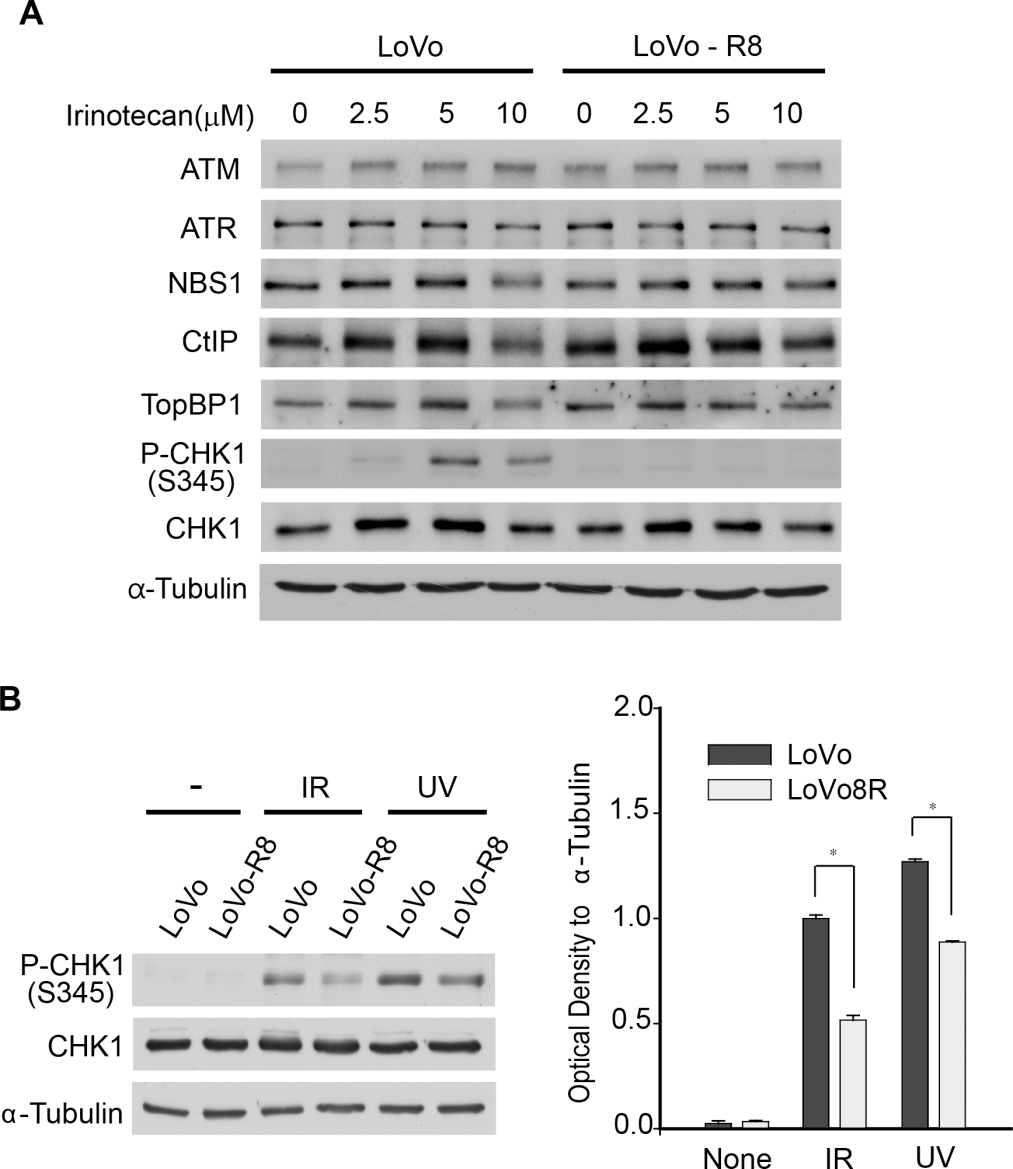
Expression patterns of genes involved in the pathway of DNA damage checkpoint. (A) Expression of several genes involved in the DNA damage response in irinotecan-resistant cells. CHK1 was activated at 5 μM in parental LoVo cells, but resistant LoVo cells did not show the activation of CHK1. (B) DNA damage such as IR and UV induced the activation of CHK1 in LoVo and LoVo-8R cells. The phosphorylation of CHK1 in response to IR (10 Gy) and UV (50 J/m^2^) was higher in LoVo cells than that in LoVo-8R cells. Graph shows the phosphorylation level of CHK1. The differences were considered significant at *p* < 0.05 by t-test. *, comparison between LoVo cells vs LoVo-R8 cells.

To find new markers related to irinotecan resistance, we investigated the expression of several genes involved in DNA damage checkpoint signaling. The irinotecan-sensitive LoVo cells showed a same levels in CtIP (CtBP interacting protein) and TopBP1 (topoisomerase (DNA) II binding protein 1) expression, in a dose-dependent manner. In irinotecan-resistant LoVo cells, CtIP expression was more or less increased. However, the expression of TopBP1 was not changed. CHK1 (CHEK1, checkpoint kinase 1) was activated by 5 μM of irinotecan in the parental LoVo cells. However, CHK1 was not activated in the LoVo-8R cells treated with the same concentration of irinotecan (Fig 3A). Consequently, only phosphorylated CHK1 showed a differential expression pattern in irinotecan-sensitive versus-resistant cells (S2 Fig). The activation of CHK1 upon exposure to ionizing radiation (IR) or UV radiation was normal in both irinotecan-sensitive and-resistant cells (Fig 3B). However, the phosphorylation level of CHK1 in response to IR and UV was higher in irinotecan-sensitive LoVo cells. These results made us further investigate cell cycle arrest-related genes, to find new markers for irinotecan resistance.

### Sestrin3 knockdown enhanced irinotecan cell cytotoxicity in resistant LoVo cells

**Fig 4:**
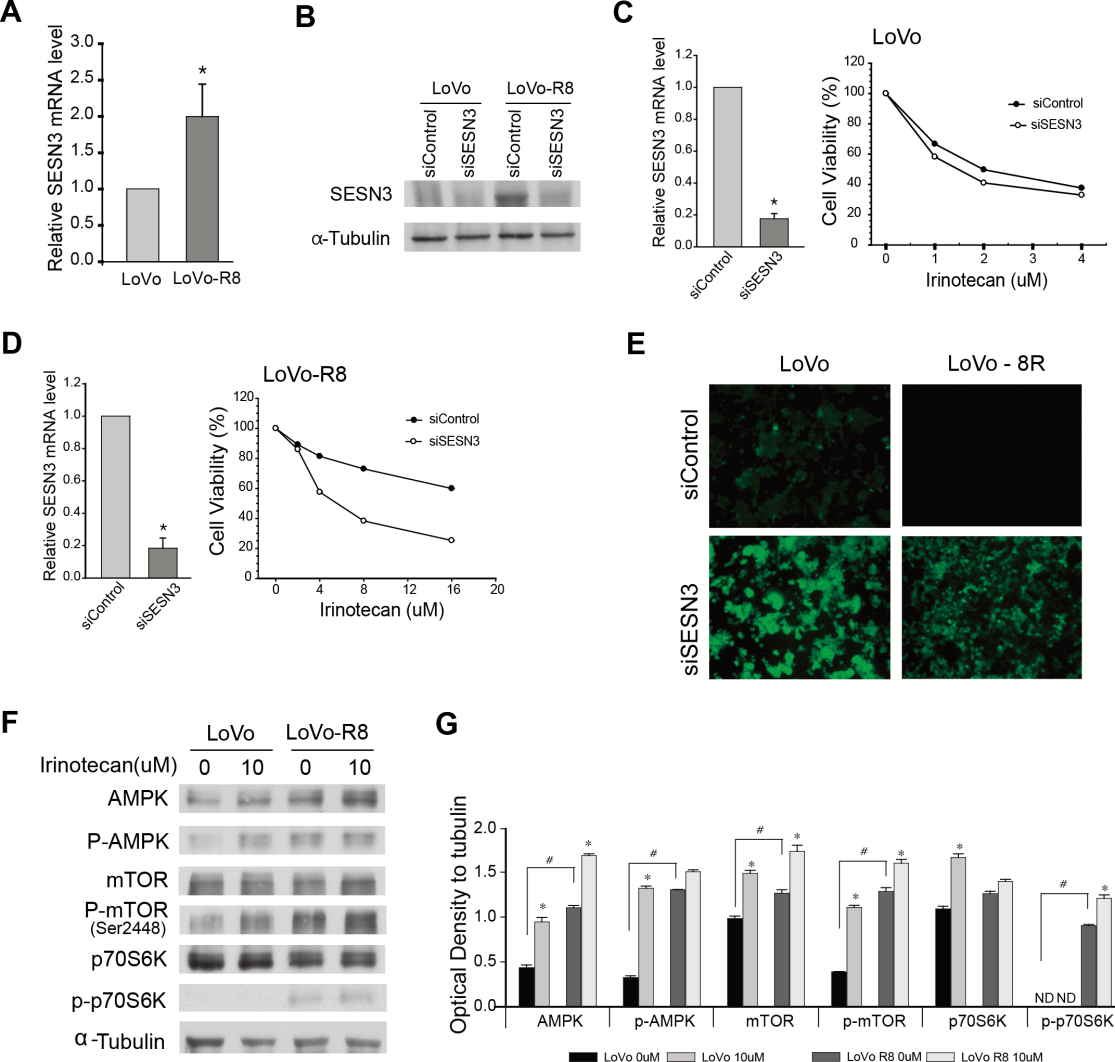
Effect of Sestrin3 knockdown on resistance to irinotecan. (A) The relative quantity of Sestrin3 mRNA level was increased in LoVo-R8 cell by realtime PCR. (B) According to upregulated Sestrin3 transcript, western blot analysis showed Sestrin3 protein level was also increased in LoVo-R8. Treatment of Sestrin3 siRNA (siSESN3) effectively down-regulated protein level of increased Sestrin3 in LoVo-R8. (C) Sestrin3 transcript was down regulated by transfecting siSESN3 in qPCR analysis (left). LoVo cells was susceptible to irinotecan with dose-dependent, and viability of LoVo was not affected even under siSESN3 treatment (right). Cells were treated with siSESN3 for 48 h. Culture media were changed with the medium containing the indicated concentration of irinotecan and further incubated for 72 h. The cell viability is presented as the percentage relative to that of untreated cells. (D) Sestrin3 transcript was also down regulated in LoVo-R8 by transfecting siSESN3 in qPCR analysis (left). LoVo-R8 cells was resistant to irinotecan, but was changed into be susceptible to irinotecan by siSESN3 treatment (right). (E) Fluorescence images of intracellular ROS stained with the CellRox green reagent. Cells were transfected with control siRNA or siSESN3. The ROS was measured after 48 h. (F) Western blot showing the protein levels of AMPK, p-AMPK, mTOR, p-mTOR, p70S6K, and p-p70S6K in LoVo and LoVo-R8 with or without irinotecan. (G) Graph showing the levels of proteins. The differences were considered significant at *p* < 0.05 by t-test. *, comparison between group without irinotecan vs group with 10 μM irinotecan in LoVo and LoVo-R8; #, comparison between LoVo vs LoVo-R8.

To find markers for irinotecan resistance, we analyzed the up-regulated genes found to be involved in cell cycle arrest, based on the microarray data. Sestrin3 was dramatically up-regulated in the LoVo-R8 cells (Table 1). Sestrin3 knockdown did not affect the toxicity of irinotecan to the parental LoVo cells. The relative quantity of Sestrin3 mRNA level was increased in LoVo-R8 cells, and western blot analysis showed Sestrin3 protein level was also increased in LoVo-R8 cells (Fig 4A and 4B). Using qPCR analysis, Sestrin3 transcript was confirmed to be down regulated by transfecting siSESN3, but the viability of LoVo cells was not affected by siSESN3 treatment (Fig 4C). Sestrin3 knockdown decreased the resistance of LoVo-R8 cells to irinotecan, reducing its IC_50_ value to 4 μM (Fig 4D). Sestrins, a family of stress-inducible proteins, share a high degree of homology with the bacterial AhpD protein that is responsible for catalyzing the reduction of peroxiredoxins. We measured ROS accumulation under controlled Sestrin3 levels in each cell line (Fig 4E). LoVo-R8 cells showed a lower ROS accumulation than their parental LoVo cells. Sestrin3 knockdown increased ROS production in both cell types.

**Table 1:** Summary of differentially regulated gene clusters of cell cycle arrest-related gene ontology in irinotecan resistant LoVo-R8 cells.

No	Gene Symbol	Gene Title	Fold change	Regulation
1	CDKN1A	cyclin-dependent kinase inhibitor 1A (p21, Cip1)	1.936	up
2	CDKN2B	cyclin-dependent kinase inhibitor 2B (p15, inhibits CDK4)	1.311	up
3	DDIT3	DNA-damage-inducible transcript 3	0.883	down
4	GADD45A	growth arrest and DNA-damage-inducible, alpha	1.085	up
5	ID2	inhibitor of DNA binding 2, dominant negative helix-loop-helix protein	0.484	down
6	IL8	interleukin 8	3.999	up
7	IRF6	interferon regulatory factor 6	0.286	down
8	PPP1R15A	protein phosphatase 1, regulatory (inhibitor) subunit 15A	1.343	up
9	PRKAB2	protein kinase, AMP-activated, beta 2 non-catalytic subunit	1.977	up
10	PSMB9	proteasome (prosome, macropain) subunit, beta type, 9 (large multifunctional peptidase 2)	2.241	up
11	PSME2	proteasome (prosome, macropain) activator subunit 2 (PA28 beta)	1.11	up
12	SESN2	sestrin 2	0.782	down
13	SESN3	sestrin 3	4.292	up
14	SOX2	SRY (sex determining region Y)-box 2	1.085	up
15	SOX4	SRY (sex determining region Y)-box 4	1.047	up
16	TGFB2	transforming growth factor, beta 2	5.426	up
17	THBS1	thrombospondin 1	0.392	down
18	TP53INP1	tumor protein p53 inducible nuclear protein 1	1.361	up

Next, we examined the phosphorylation of AMPK, mTOR, and p70S6K to investigate whether Sestrin3 affects resistance to irinotecan through the AMPK/TORC1 pathway (Fig 4F and 4G). LoVo-R8 cells showed higher expression levels of AMP activated protein kinase (AMPK) and of AMPK phosphorylation than parental LoVo cells. Phosphorylation of mTOR and S6K (p70S6 kinase), an mTORC1 substrate was increased in LoVo-8R cells. Moreover, LoVo-8R cells showed constitutive activation of AMPK and mTOR with or without irinotecan compared with LoVo cells.

## Discussion

Irinotecan has been widely evaluated as a single agent as well as in combination regimens with other chemotherapeutic drugs. After approval for treatment of metastatic colorectal cancer, irinotecan has been used to treat a number of other cancers, including lung cancer, gastric cancer, brain tumors, and breast cancer. However, drug resistance of tumor to irinotecan has limited its efficacy in clinical therapies. We established a stable cell line that is resistant to irinotecan. The most sensitive cell line was selected from eight colorectal cell lines, based on cytotoxicity assays. We induced chronic resistance by exposing cells to an increasing dose of irinotecan. To confirm their adaptation, cells that acquired resistance were cultured in a drug-free medium for three passages. After culturing in the drug-free medium, the LoVo-R8 cells that had acquired resistance to irinotecan maintained it to the same level as the freshly established cells. The microarray data were analyzed to identify key genes that play important roles in the resistance to irinotecan. A total of 1165 differentially expressed genes (DEGs) were obtained. A Gene Ontology enrichment analysis revealed that the level of Sestrin3 was dramatically increased. However, the expression levels of the other two members of the Sestrin family, Sestrin1 and Sestrin2, were slightly changed. Sestrins are a family of highly conserved, stress-responsive proteins. Sestrin1 and Sestrin2 are transcriptionally regulated by p53. Sestrin3, regulated by forkhead transcription factor, exhibits oxidoreductase activity *in vitro*. It has been reported that SESN3 can protect cells against oxidative stress by modulating peroxiredoxin (Prx) regeneration [10]. We expected that Sestrin1 and Sestrin2 expression would be up-regulated because various DNA damage agents are known to affect cell cycle progression through p53 activation. From our array data, S2 Table presents the expression levels of Sestrins as well as the genes relating to the p53 pathway. Of the 24 genes identified in this study as being involved in the irinotecan pathway of colorectal cancer, the expression levels of *CES1* and *CES2* (involved in the conversion of the pro-drug to irinotecan) were diminished by approximately 25%. Of the 24 genes, the ones encoding ATP binding cassette (ABC) proteins involved in efflux, such as *ABCB1* and *ABCG2*, were the most highly up-regulated genes. Resistance to irinotecan is affected by DNA repair systems. *ERCC1* and *ERCC2*, which participate in nucleotide excision repair, showed some increase in their expression. However, the expression of *MLH1* and *MLH6* (involved in the mismatch repair process) changed very little. Of the 24 genes, those involved in the apoptotic pathway did not show any difference in expression between the irinotecan-sensitive and irinotecan-resistant cells. Considering these variations in gene expression, the acquired resistance to irinotecan in LoVo-R8 cells might be due to diminished cellular accumulation of irinotecan, or to conversion of irinotecan to an active metabolite because SN-38 was decreased to some extent.

Irinotecan treatment is associated with G2 cell cycle arrest. We investigated the category of cell cycle arrest in Gene Ontology, to find the key targets of cell cycle-mediated resistance. Sestrin3 was shown to modulate Prx regeneration in cancer cells. The expression level of Sestrin3 was increased through transcriptional activation by FOXO1. While the Sestrin family is implicated in redox control [5, 8, 12], recent studies have demonstrated the role of Sestrins in mTORC1 signaling. mTORC1 is inhibited by members of the Sestrin family (SESN1/2), through AMPK-mediated activation of the TSC1/2 complex [12]. Sestrin3 has been reported to hinder mTORC1, based on the finding that FOXO1/3a-mediated transcriptional up-regulation of Sestrin3 proceeds to activate AMPK/TSC1/2, which eventually inhibits mTORC1 activity [13]. Additionally, mTORC1 activation leads to the accumulation of ROS, resulting in the activation of JNK/FOXO signaling, and a positive feedback loop of Sestrin expression [5, 14]. However, the role of Sestrin3 in oncogenesis and resistance to cancer drugs remains ambiguous. Examination of AMPK/mTOR pathway in LoVo and LoVo-R8 cells showed that AMPK signal was activated in irinotecan treated cells or irinotecan-resistant cells (Fig 4F). Generally, it is expected that mTOR signaling under inhibition by active AMPK would induce autophagy[15]. In our study, however, AMPK activation did not inhibit mTOR. Although mTOR signaling is closely associated with AMPK signal and is in fact down-stream to AMPK, TSC1/2 and Rheb are hub molecules of mTOR signaling pathway integrating diverse inputs [16, 17]. Our study reveals a positive rather than a negative regulatory relationship between AMPK and mTOR.

Our results and interpretations were focused on the antioxidant effect of Sestrin3 on resistance to cancer drugs. In general, cancer cells undergo aerobic glycolysis to satisfy the high nutrient demand imposed on the cellular and extracellular environment by their rapid proliferation. Demands for increased energy production bring cancer cells to face severe oxidative stress, which activates various defense and repair mechanisms against genotoxic drugs, although ROS may also function as specific second messengers in signaling cascades involved in cell proliferation and differentiation. In cancer cells, the accumulation of ROS might stimulate cellular proliferation, mutagenesis, and genomic instability [18, 19]. Accordingly, an accumulation of large amounts of ROS is detected in cells transformed with the *RAS* oncogene [18, 20, 21]. Although the mechanism underlying ROS overproduction in response to Ras is not well characterized, Sestrins have been demonstrated to participate in this process [22]. When H-Ras and N-Ras are converted to their respective activated forms, the *Sestrin1* and *Sestrin3* genes are negatively regulated. The inhibited expression of Sestrins causes an oxidative burst, and increased levels of DNA oxidation and chromosomal instability [22]. Sestrin1 and Sestrin3 in Ras-transformed cells prevent the accumulation of ROS and reduces the oxidative DNA damage. LoVo cells have K-Ras mutation. Oncogenic ras mutation brings the repression of Sestrin genes and up-regulation of reactive oxygen species [22]. Our results showed that irinotecan resistant LoVo cells with high level of Sestrin3 had lower ROS accumulation compared to irinotecan sensitive LoVo cells. Sestrin3 knockdown caused high levels of ROS accumulation, which led to severe cytotoxic effects. Cancer has similar mechanisms to those in degenerative diseases, in that an elevated oxidative level can contribute to carcinogenesis involving transformation, genomic instability, invasiveness, and angiogenesis [18]. Mice deficient in antioxidant enzymes are reported to be prone to cancer [23].

As shown in our study, the antioxidant Sestrin3 is involved in resistance to the drug irinotecan. Our results provide useful information for the identification of different biochemical targets to overcome resistance to irinotecan, and for the prediction of the response to irinotecan chemotherapy.

## Supporting Information

S1 Fig Transcriptome analysis. Hierarchical cluster analysis of the gene expression of the irinotecan-resistant LoVo cell line. For the DNA microarray, total RNAs were isolated from established LoVo cells, LoVo 8R- or 8R+ designating irinotecan addition to the culture medium. We used the LoVo 8R- data in this experiment.
(TIF)

S2 Fig Quantitation of protein expression levels of DNA damage response genes. Protein bands from immunoblots in Fig 3 were scanned and quantitated. α-Tubulin bands were used for normalization. One-way ANOVA with Tukey’spost-hoc test was carried out to examine the statistical significance. Asterisks indicate *P* < 0.05.
(TIF)

S3 Fig Effect of Sestrin3 on toxicity of irinotecan in non-tumor colon cell line. (A) Sestrin3 knockdown efficacy. Cells were transfected with Sestrin3 siRNA (30 nM), total RNA was prepared 48 h later, and quantitative PCR was performed using Power SYBR Green Cells to Ct kit (Ambion). (B) Cytotoxicity assay of irinotecan by CCK-8 kit.
(TIF)

S4 Fig FACS analysis of cellular ROS contents. Cells were transfected with siSESN3 for 48 h and then added with irinotecan for 24 h. CellROX reagent (A) for measurement of total cellular ROS contents or MitoSOX (B) for measurement of mitochondrial superoxide was added to final concentration of 5 μM according to the manufacturer’s recommendation. Cells were exposed to reagents for 30 min and washed twice with Hank’s Buffered Salt Solution (HBSS) containing calcium and magnesium. For flow cytometry analysis, 30 min after applying MitoSOX, cells were trypsinized and were washed with HBSS. Cells were fixed with 4% formaldehyde PBS solution, and flow cytometry was performed at excitation/ emission of 488/530 (CellROX) or 510/580 nm (MitoSOX).
(TIF)

S1 Table Gene expression of 24 genes in the irinotecan pathway. (TIF)

S2 Table Expression of genes in the p53 pathway, based on a KEGG pathway analysis. (TIF)
