# Supplementary figures and images for: Identification of Sestrin3 Involved in the In vitro Resistance of Colorectal Cancer Cells to Irinotecan
Source: PLoS One. 2015 May 14;10(5):e0126830. doi: 10.1371/journal.pone.0126830 (PMC4431826; doi:10.1371/journal.pone.0126830)

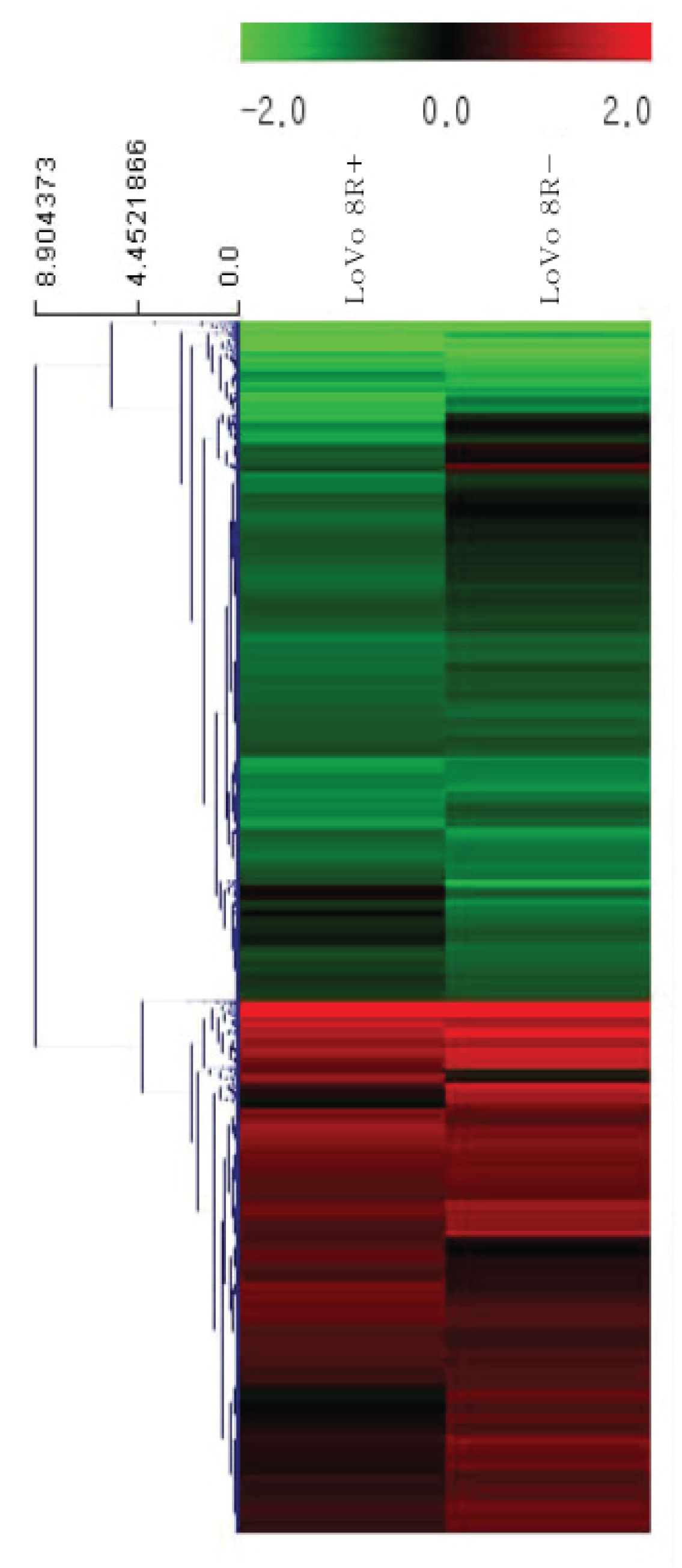

Supplement: S1 Fig — Hierarchical cluster analysis of the gene expression of the irinotecan-resistant LoVo cell line. For the DNA microarray, total RNAs were isolated from established LoVo cells, LoVo 8R- or 8R+ designating irinotecan addition to the culture medium. We used the LoVo 8R- data in this experiment. (TIF) [file pone.0126830.s001.tif]

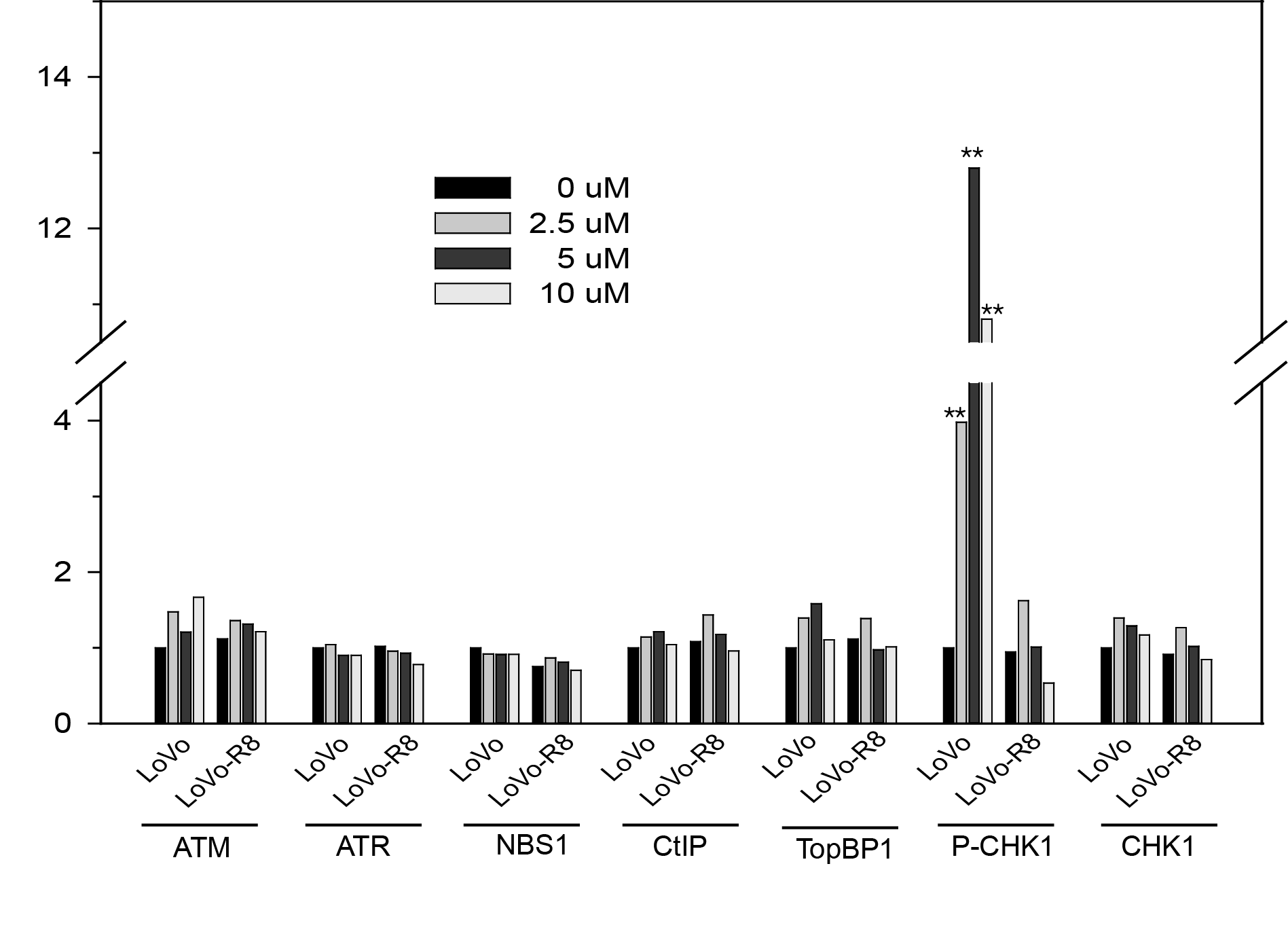

Supplement: S2 Fig — Protein bands from immunoblots in Fig 3 were scanned and quantitated. α-Tubulin bands were used for normalization. One-way ANOVA with Tukey’spost-hoc test was carried out to examine the statistical significance. Asterisks indicate P < 0.05. (TIF) [file pone.0126830.s002.tif]

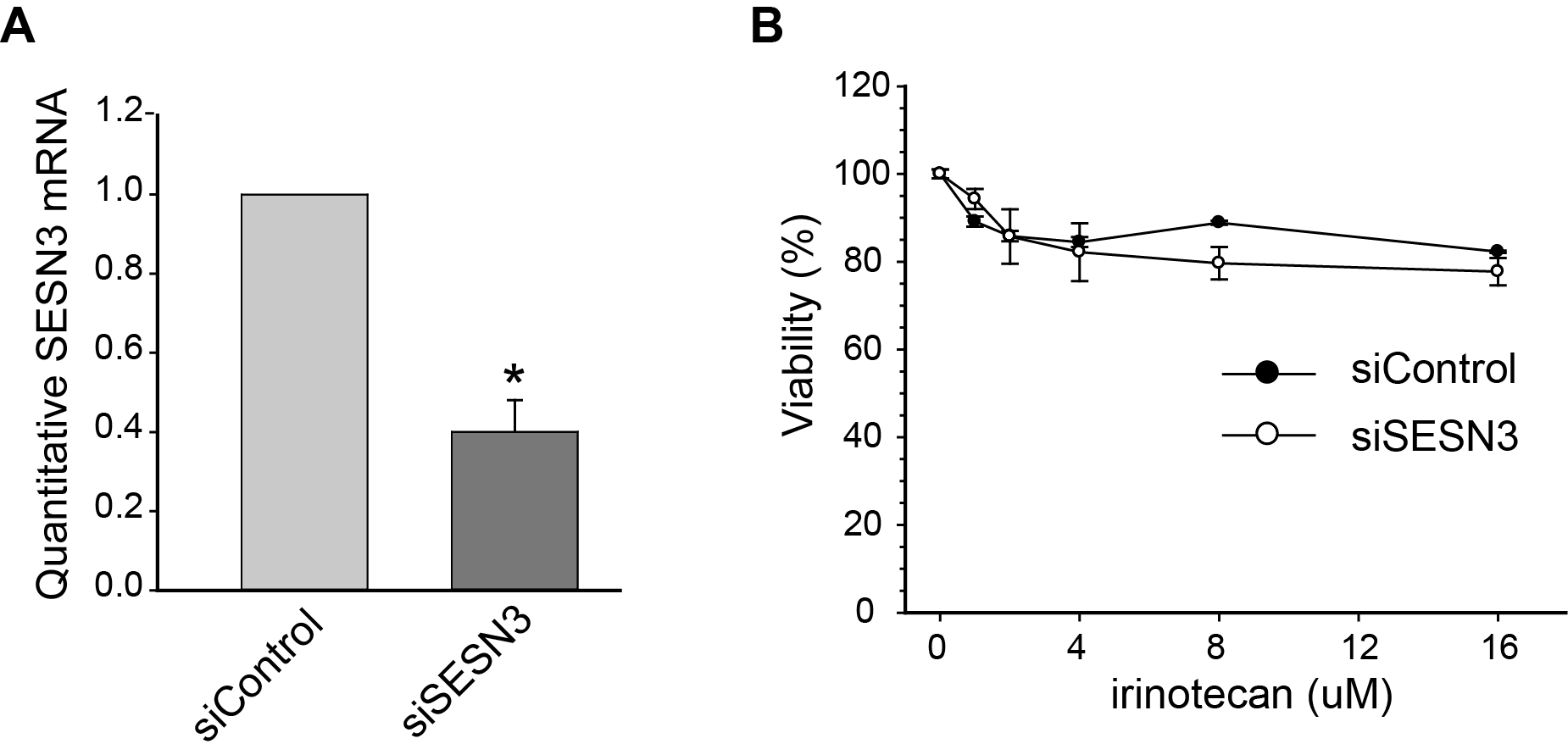

Supplement: S3 Fig — (A) Sestrin3 knockdown efficacy. Cells were transfected with Sestrin3 siRNA (30 nM), total RNA was prepared 48 h later, and quantitative PCR was performed using Power SYBR Green Cells to Ct kit (Ambion). (B) Cytotoxicity assay of irinotecan by CCK-8 kit. (TIF) [file pone.0126830.s003.tif]

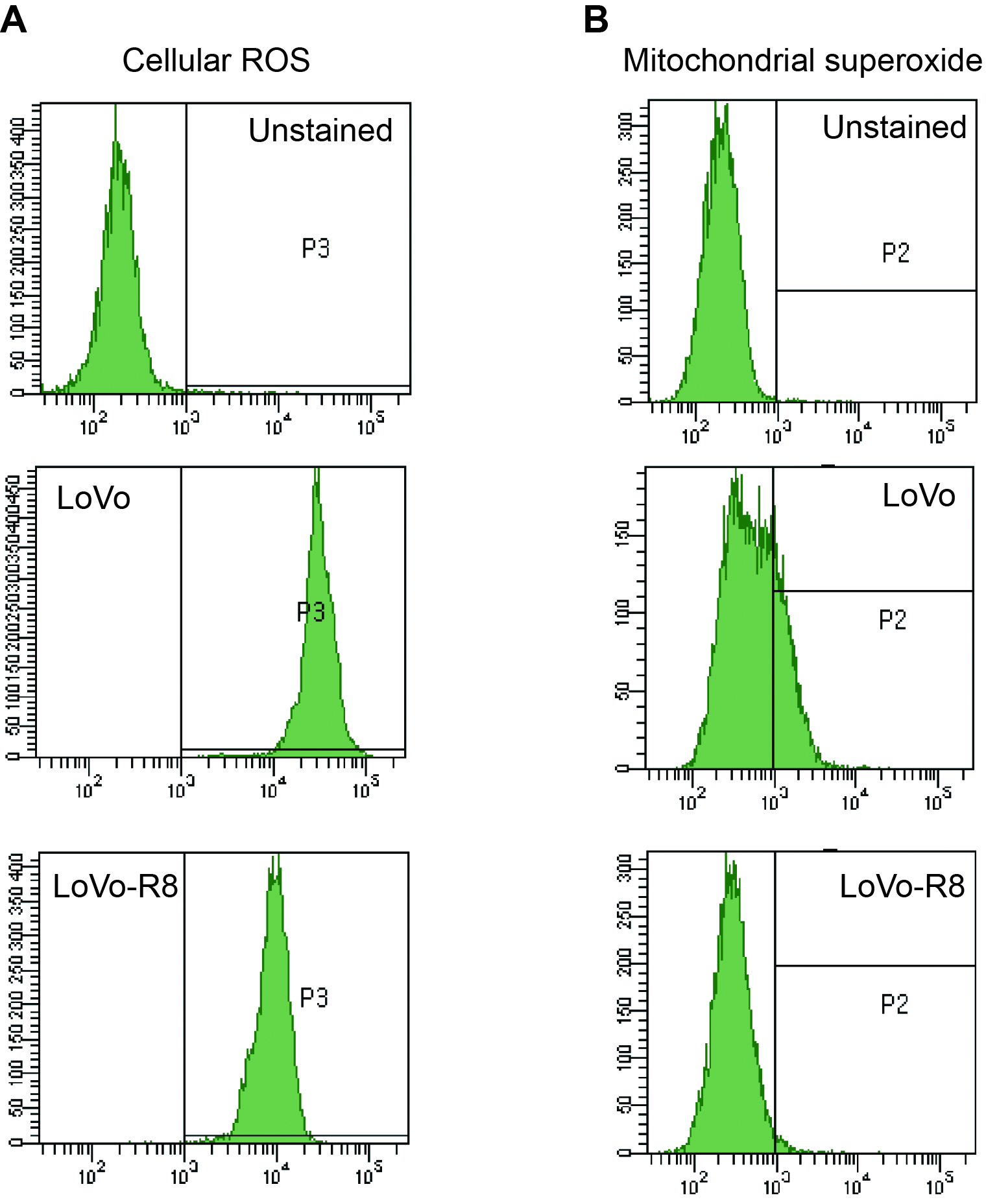

Supplement: S4 Fig — Cells were transfected with siSESN3 for 48 h and then added with irinotecan for 24 h. CellROX reagent (A) for measurement of total cellular ROS contents or MitoSOX (B) for measurement of mitochondrial superoxide was added to final concentration of 5 μM according to the manufacturer’s recommendation. Cells were exposed to reagents for 30 min and washed twice with Hank’s Buffered Salt Solution (HBSS) containing calcium and magnesium. For flow cytometry analysis, 30 min after applying MitoSOX, cells were trypsinized and were washed with HBSS. Cells were fixed with 4% formaldehyde PBS solution, and flow cytometry was performed at excitation/ emission of 488/530 (CellROX) or 510/580 nm (MitoSOX). (TIF) [file pone.0126830.s004.tif]

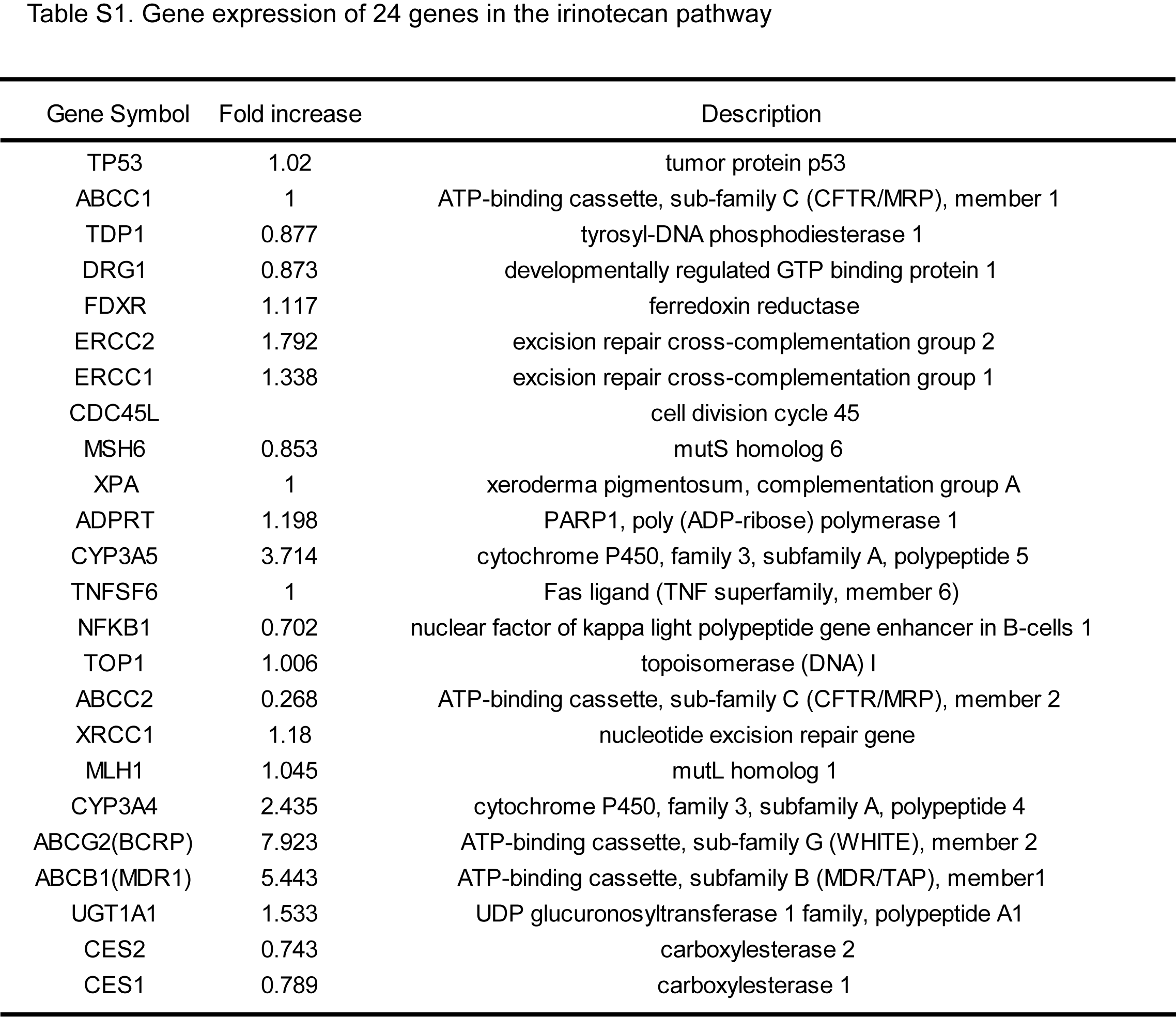

Supplement: S1 Table — (TIF) [file pone.0126830.s005.tif]

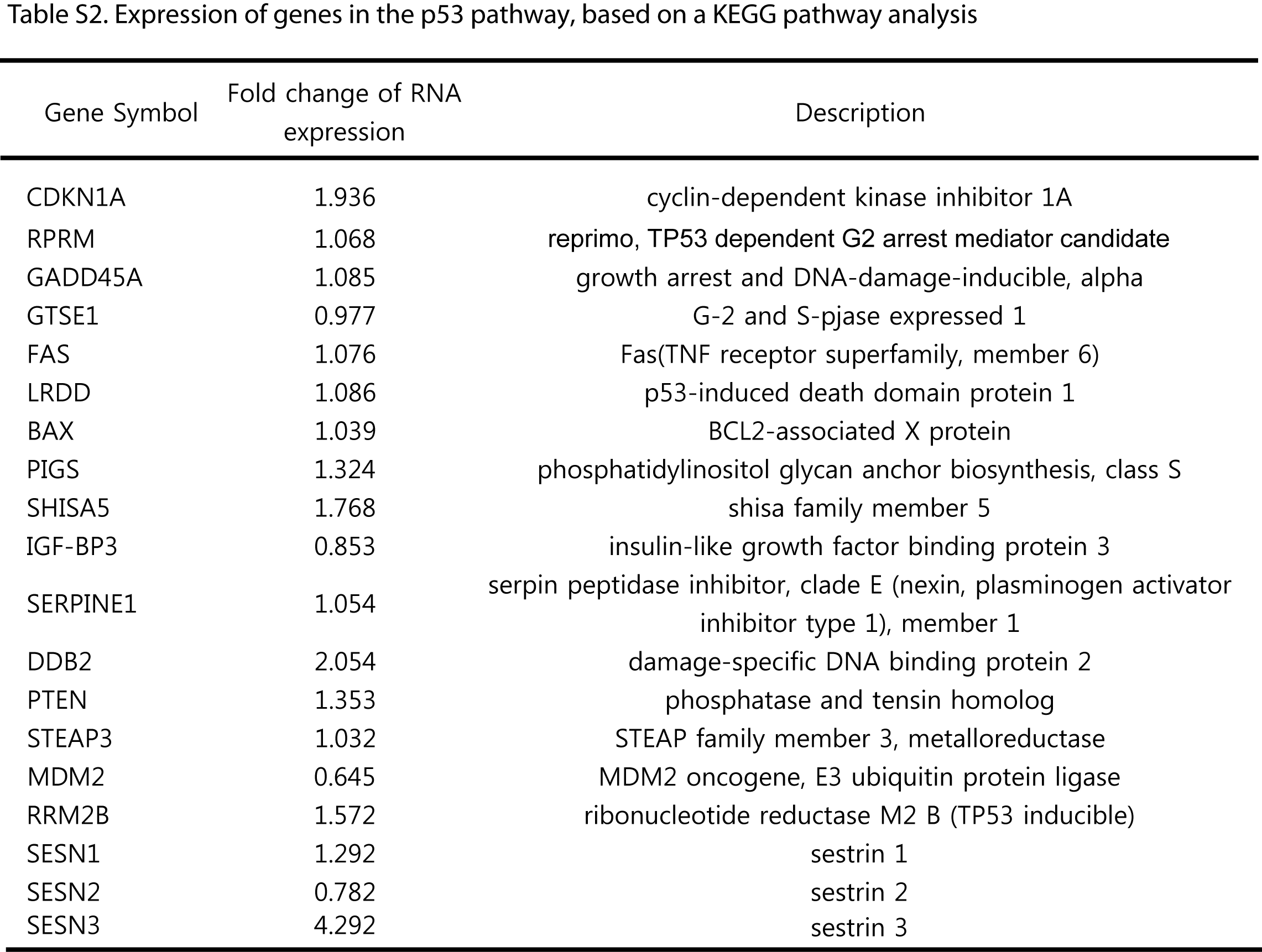

Supplement: S2 Table — (TIF) [file pone.0126830.s006.tif]
